# Mobile genetic elements in *Clostridium difficile* and their role in genome function

**DOI:** 10.1016/j.resmic.2014.12.005

**Published:** 2015-05

**Authors:** Peter Mullany, Elaine Allan, Adam P. Roberts

**Affiliations:** Department of Microbial Diseases, UCL Eastman Dental Institute, University College London, 256 Gray's Inn Road, London WC1X 8LD, UK

**Keywords:** Conjugative transposon, PaLoc, Bacteriophage, Horizontal gene transfer, Tn*5397*, Tn*916*

## Abstract

Approximately 11% the *Clostridium difficile* genome is made up of mobile genetic elements which have a profound effect on the biology of the organism. This includes transfer of antibiotic resistance and other factors that allow the organism to survive challenging environments, modulation of toxin gene expression, transfer of the toxin genes themselves and the conversion of non-toxigenic strains to toxin producers. Mobile genetic elements have also been adapted by investigators to probe the biology of the organism and the various ways in which these have been used are reviewed.

## 1 Introduction

Approximately 11% of the *Clostridium difficile* genome is made up of mobile genetic elements (MGEs) [1] which provides the bacterium with a remarkable genetic plasticity. However the exact role of the mobile genome in the organism's biology, evolution and pathogenicity is only now beginning to be understood.

MGEs are highly heterogeneous and here we will define them in the loosest possible terms as any region of nucleic acid that can move from one part of a genome to another or between genomes. By this definition, MGEs range from the simple insertion sequences which contain only the genetic information required for movement from one part of the genome to another, to bacteriophage, large conjugative transposons and mega plasmids which are complex genomes in themselves. In this review we will provide examples of some of these genetic elements and discuss the mechanisms of horizontal gene transfer and the consequent biological effects on the host cell.

## 2 Plasmids

Plasmids are extra chromosomal genetic elements which range in size from very small, around 1.5 kb to mega plasmids which can reach the size of bacterial genomes e.g. [2]. In *C. difficile,* plasmids have been identified in several different strains [1,3–5]. To our knowledge no phenotype has been found to be conferred on *C. difficile* by a naturally occurring plasmid. However, naturally occurring *C. difficile* plasmids have been used to generate vectors for the genetic manipulation of the organism [6,7].

The plasmids used as the basis for *C. difficile* genetic manipulation are small and cryptic and contain only the genetic information required for their replication and maintenance. The subsequent addition of *Escherichia coli* origins of replication and origins of transfer allowed the construction of shuttle vectors that can replicate in both *C. difficile* and *E. coli* and can be mobilised from *E. coli* to *C. difficile* [6,7]. Vectors based on these cryptic *C. difficile* plasmids have been extensively modified to provide a range of research tools for the bacterium [8,9].

## 3 IS sequences

These are simple MGEs, containing just the genetic information required to translocate from one part of the genome to another. However IS elements in *C. difficile* have not been investigated to date and they will not be discussed further here. However IStrons which are a hybrid element consisting of an IS element and a group I intron are discussed below.

## 4 The mobile introns

Introns were first discovered in 1977 when it was found that eukaryotic genes were split, i.e. that the coding region was interrupted by non-coding sequences (introns) that were spliced out of the RNA transcripts before being translated [10,11]. At the time this was a surprising finding that represented a paradigm shift in our understanding of biology. Initially it was thought that bacteria and their viruses did not contain introns however this notion was shown to be incorrect with the finding of an intron in a phage genome [12]. Subsequently introns have been found in many different bacteria, nearly always found in association with mobile DNA, although they are rare in prokaryotes compared to eukaryotes. Bacterial introns are classified into either group I or group II according to their conserved secondary structure [13].

### 4.1 Group II introns

The first intron found in *C. difficile* was a group II intron and it was contained within *orf14* of the conjugative transposon, Tn*5397* [14]. Group II introns often contain an ORF encoding a multifunctional protein made up of maturase, endonuclease and reverse transcriptase domains. This multi-functional protein becomes part of a riboprotein complex in conjunction with the intron-encoded RNA. This riboprotein complex is required for effective splicing and the riboprotein can catalyse intron transposition [13]. Work in our laboratory has shown that the *C. difficile* intron is capable of splicing and that the intron-encoded protein is required for this process [15]. Transposition of this element has not been demonstrated, but group II introns have been found in other *C. difficile* elements suggesting that they are capable of transposition [16].

The contribution of the mobile group II introns to *C. difficile* biology has not been determined but, as one has been shown to splice perfectly from its host gene [15] and has a small size, their biological costs are likely to be low. Group II introns may have a role in regulation as differential splicing can allow alternative proteins to be produced. This has not been demonstrated in *C. difficile* but in *Clostridium tetani,* a group II intron has been characterised and shown to undergo alternative splicing to produce different isoforms of surface layer proteins [17]. Group II introns have been used in biotechnological approaches forming the basis of the TargeTron which has been used for making targeted gene knock-outs [18]. TargeTrons adapted for the clostridia have been termed ‘clostrons’ [8].

### 4.2 IStrons

IStrons are hybrid genetic elements combining a group I intron and an insertion sequence [19]. The whole element is capable of both splicing out of primary transcripts and transposing to new genomic sites. These elements are relatively wide-spread in *C. difficile.* Analysis of the splicing reaction in IStrons has shown that they can have alternative splice sites leading to the production of variant proteins [19]. As IStrons have been found inserted into the *C. difficile* toxin A gene, alternative splicing could produce alternative toxins [19], although IStron mediated toxin variation has not yet been demonstrated.

## 5 Conjugative and mobilisable transposons

Conjugative transposons (CTns) also called Integrative Conjugative Elements (ICE), are genetic elements that are capable of transferring themselves from a donor cell to a recipient using a conjugation-like mechanism. Unlike plasmids these elements do not normally contain an origin of replication so in order to survive they must be integrated into a replicon. Mobilisable transposons (MTns) can also be transferred by a conjugation-like mechanism but these elements lack the genes required for conjugation and use those of CTns or conjugative plasmids that are present in the same cell. For a detailed discussion of the properties of these types of elements, the reader is referred to the following recent reviews [20–23]. Here we will discuss what is known about the CTns and MTns from *C. difficile.*

Several DNA sequencing projects have shown that *C. difficile* contains a plethora of putative CTns and MTns and some of these are proven to be active and capable of conjugative transfer [1,16,24,25]. The typical genetic organisation of these elements is that they contain regions required for integration and excision, conjugation (in the case of CTns, this is absent in MTns), regulation and an accessory region. The latter usually contains genes which are thought to allow the bacterium to survive in particularly challenging environments, such as antibiotic resistance genes and ABC transporters [16]. Some CTns have been shown to encode sigma factors; whether these are required for regulation of the CTn itself or have a more global role in the host biology is not known.

Surprisingly, despite the large number of CTns and MTns detected in different strains of *C. difficile,* very few have been studied in detail. Although bioinformatics analysis has led to predictions of how these elements might affect the biology of *C. difficile*, there is little experimental evidence. The following section will discuss what is known about how these elements affect the biology of their host.

### 5.1 Tn*5397* and Tn*916*

Tn*5397* is the best understood of the *C. difficile* CTns. It was originally discovered in strain 630 where it conferred tetracycline resistance [14,26,27]. It is capable of transferring between *C. difficile* strains and to and from *Bacillus subtilis* and *Enterococcus faecalis* and the element has also been found in *Streptococcus* spp. [27–29]. The element is 21 kb in size and is very closely related to Tn*916,* the paradigm for this family of MGEs [30]. The ends of the elements are very different and Tn*5397* encodes a large serine recombinase, TndX, responsible for integration and excision [31]. TndX directs the element into two highly preferred insertion sites in *C. difficile* R20291 [32]. However in *B. subtilis* there are no obvious preferred insertion sites apart from a central GA dinucleotide which is always present in the target site.

Both Tn*916* and Tn*5397* confer tetracycline resistance on *C. difficile* but in contrast to Tn*916*, which inserts into multiple regions of the genome (see below), Tn*5397* inserts into DNA predicted to encode a domain initially termed Fic (filamentation processes induced by cAMP). This was characterised in *E. coli* where mutation of the *fic* gene resulted in filamentous growth [33]. Latter work showed that Fic domains are ubiquitous in all domains of life and they can be present in a myriad of different proteins, reviewed in [34]. Proteins containing Fic domains are involved in post-translational modification of their targets via UMPylation, AMPylation, phosphorylation, or phosphocholination [34]. In order to determine if the sequence of the *C. difficile* Fic encoding domain itself is responsible for the remarkable targeting of this region by Tn*5397* we cloned it into *B. subtilis* and showed that Tn*5397* always entered the genome in the cloned sequence encoding the Fic domain, demonstrating that if this region is present it is used by Tn*5397* [32]. Band shift assays showed that this was most likely a result of TndX showing preferential binding to the sequence in the Fic encoding domain [32]. It is intriguing that TndX has evolved to target Fic encoding domains; perhaps because these are ubiquitous in all forms of life they provide a suitable target for a promiscuous CTn.

Tn*916* has also been found in *C. difficile* and transferred into this organism from *B. subtilis* [35]. In contrast to Tn*5397,* Tn*916* contains genes encoding a tyrosine recombinase and an excisionase which are responsible for integration and excision of the element. This difference results in Tn*916* having the ability to insert into multiple sites in the *C. difficile* genome although it does have a preferred consensus site which is present in the *C. difficile* genome 10^5^ times [35]. This consensus is present more times in intergenic regions than within ORFs [35].

Tn*916* has also been used as a tool for the investigation of *C. difficile* biology, both for mutagenesis [35] and for gene cloning in the organism [7,36,37]. As a tool for mutagenesis, the site preferences noted above for the element is a disadvantage. Another system for *C. difficile* transposon mutagenesis has been developed based on a Mariner transposon and it is likely that this system will be the mutagenesis tool of choice as it does not appear to suffer from the site specificity of Tn*916* [9]. However, since the first report of a *C. difficile* Mariner system in 2010, no large scale mutagenesis studies have been reported suggesting that Tn*916* may have to be revisited as a tool for mutagenesis.

Tn*916* was the first genetic element used for successful cloning in *C. difficile* and it made use of the fact that cargo DNA can be added to the region of Tn*916* upstream of the *tet*(M) gene without affecting its conjugative ability. A vector that contained regions of homology to Tn*916* was designed and this was transformed into *B. subtilis* and the recombinant transposon could be transferred by conjugation to *C. difficile* [36]. Cloning into Tn*916* has been used to investigate the role of the *virR* [7] cwp66 [37] and the skin element (see below) [38].

### 5.2 Tn*4453*a and Tn*4453*b

**Fig. 1:**
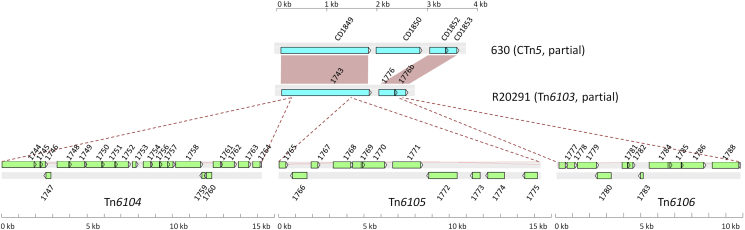
Comparison of part of CTn5 from *C. difficile* 630 to the part of Tn*6103* that contains the three putative MTns. The red boxes represent homologous genes between CTn*5* and Tn*6103*. The points of insertion of the three putative MTns are represented by the dotted lines. (Figure reproduced from; Brouwer MSM, Warburton PJ, Roberts AP, Mullany P, Allan E. (2011) Genetic organisation, mobility and predicted functions of genes on integrated, mobile genetic elements in sequenced strains of *Clostridium difficile*. PLoS ONE 6(8): e23014. http://dx.doi.org/10.1371/journal.pone.0023014).

Tn*4453a* and Tn*4453b* were initially found in *C. difficile* strain W1 where they conferred resistance to chloramphenicol [39]. These two elements only differ from each other by a few base pairs and are very closely related to the *Clostridium perfringens* MTn, Tn*4451* [40]. When Tn*4451* was cloned in *E. coli* it could be mobilised by plasmid RP4 to *C. perfringens* [41]. Although the *C. difficile* MTns could not be transferred in the laboratory, the fact that they are very closely related to a proven MTn suggests that they are likely to be capable of mobilisation. Genetic elements related to Tn*4453* and Tn*4451* have been found contained within the large *C. difficile* CTn, Tn*6103* [16] Fig. 1. One of these elements, Tn*6104,* could excise from Tn*6103*. Unlike Tn*4453* which contains the chloramphenicol resistance gene *catD,* Tn*6104* has genes predicted to encode a transcriptional regulator, a two component regulatory system, an ABC transporter, three sigma factors and a putative toxin-antitoxin system [16]. The role of these genes in *C. difficile* biology remains to be determined.

### 5.3 Tn*5398*

Tn*5398* was originally discovered because it was responsible for transferable macrolide, lincomycin, streptogramin B (MLS) resistance, encoded by the *erm*(B) gene [42,43]. The element was capable of transfer between *C. difficile* and *B. subtilis* [42] and between *C. difficile* and *Staphylococcus aureus* [43]. Comparison of the sequences in transconjugant and recipients, allowed the ends of the element to be determined showing that it was 9.6 kb in length [44]. Tn*5398* is a rather unusual element as it does not contain any obvious recombinases and no circular forms have been found. However the element does contain putative origins of transfer (*oriT*) sites. There are at least two ways, which are not mutually exclusive, in which Tn*5398* could transfer. One of the CTns present in the donor strain could provide integration/excision functions to generate a circular intermediate molecule which is then nicked at the *oriT* site and transferred to a recipient using the conjugation functions of a CTn; integration in the recipient could either be by homologous recombination or by using a site-specific recombinase in the recipient. The other possibility is that the element is not excised but that a portion of the genome is transferred to the recipient and integrates into the recipient chromosome by homologous recombination. This process is analogous to high frequency recombination (hfr) seen when the *E. coli* F-factor is integrated into the chromosome and mobilises transfer of the whole chromosome to a suitable recipient. In this case, one of the integrated CTns could be mediating the transfer; this would require that the mobilising CTn does not excise. We have proposed that this is the mechanism responsible for transfer of the PaLoc, see below. It should be noted that the term hfr for these types of transfer events is confusing as they are not high frequency recombination events; we propose that the term chromosomal transfer and recombination (CTaR) is used instead.

A genetic element that also encodes resistance to MLS antibiotics mediated by an *ermB* gene has been found in *C. difficile*. This element, Tn*6194,* has a conjugation region that is closely related to that of Tn*916* but contains an accessory region that is related to Tn*5398* [45]. This modular composition is commonly observed amongst the MGEs in *C. difficile*. A Tn*6194*-like element has been shown to be capable of transfer between *C. difficile* strains and to *E. faecalis* [45].

## 6 The *skin*^Cd^ element

The sigK intervening sequence (skin) element was originally identified as a prophage-like sequence integrated into the *B. subtilis sigK* gene [46]. When *B. subtilis* sporulates, the element excises only in the mother cell, resulting in an intact *sigK* gene and production of the sigma factor K which is required for the sporulation cascade. *C. difficile* also contains a skin element, skin^Cd^ integrated into the *sigK* gene [38]. In common with *B. subtilis,* excision of this element in the mother cell is required for sporulation [38]. In contrast to *B. subtilis,* however, *C. difficile* strains that do not contain the skin element sporulate very poorly. Skin^Cd^ is much smaller than the *B. subtilis* skin element at 14 kb and appears to be degenerate, only retaining its site-specific recombinase (required for excision in the mother cell). A plausible scenario for the evolution of skin^Cd^ is that a prophage entered the *sigK* gene in an ancestral *C. difficile* strain and became adapted to sense the cellular stress that is a prelude for sporulation (and the phage lytic cycle) so that it excised and entered the lytic cycle. It must also have had the ability to avoid excision from the genome destined for the spore so that the prophage genome was maintained. Further evolutionary events may have resulted in the element losing the ability to kill the host and further integration into the host cell's physiology.

The exact role of skin^Cd^ has not been determined but it may be involved in the correct timing of SigK production [47]. However the regulatory signal that induces skin^Cd^ to excise has not been determined.

## 7 Bacteriophages

*C. difficile* has been shown to contain a number of different bacteriophages; for a recent review see Hargreaves and Clokie [48]. Three of these phages, ϕCD119, ϕCD38-2 and ϕCD27 are able to up- and down-regulate production of the toxins [49–51]. In the case of ϕCD119, this occurs by direct binding of a regulatory protein to the region upstream of *tcdR* within the PaLoc which encodes a positive regulator of toxin production [49].

The phage ΦC2 has been shown to mediate the transduction of an MLS resistance gene contained within an MTn Tn*6215* [52]. This is the first example of transduction in *C. difficile* demonstrating that phage have a role in mediating the spread of antibiotic resistance and potentially in the spread of other genes.

Phage particles have been found in the stools of patients infected with *C. difficile* demonstrating that lytic phage is produced during infection. Identical phages were found within the genome of the infecting strain, indicating that the phages were induced within the patients [53]. These workers also showed that lysogens of these phages could be induced to produce viral particles by treatment with sub-inhibitory concentrations of ciprofloxacin, moxifloxacin, levofloxacin, and mitomycin C suggesting that administration of these antibiotics to *C. difficile*-infected patients will promote phage mobility.

Phages also have the potential to be used as therapy for *C. difficile* associated disease. They are considered particularly attractive in this regard as they will specifically kill *C. difficile* without collateral damage to the microbiota. Phage ϕCD27 has been evaluated in a gut ecosystem model where it showed promise in treatment of a model infection compared to an untreated control [54]. However, for phages to be safely used, we need to understand more about their biology to avoid unwanted side effects like the transduction of antibiotic resistance genes and the up-regulation of toxin genes. Another problem encountered with the development of phage as therapeutic agents for *C. difficile* infection, is that all of those investigated to date form lysogens which would prevent efficient killing of all the infecting bacteria.

## 8 Transfer of the PaLoc

The PaLoc contains the genes encoding toxins A and B, the major virulence factors of the organism [55]. In addition to these genes, it contains a gene encoding a positive regulator of toxin gene expression, *tcdD* [56], a homologue of a phage holin, *tcdE*, and *tcdC* (see below). There is some controversy in the role of TcdE in toxin release and its exact role requires further experiments [57,58]. TcdC was initially proposed to be a negative regulator of toxin production but recent genetic evidence does not support this idea [59,60]. The PaLoc has some of the properties of a MGE as it occupies the same genomic location in all toxigenic strains and in non-toxigenic strains; it is replaced with 115 bp of non-coding sequence [55]. The phage-like holin gene and regulatory interaction with various phage also implies a relationship with MGEs [49,50]. However the element does not contain an obvious *oriT*, nor does it contain any predicted recombinase genes. Despite these observations, we have shown that the PaLoc is capable of transfer from toxigenic to non-toxigenic strains [61].

**Fig. 2:**
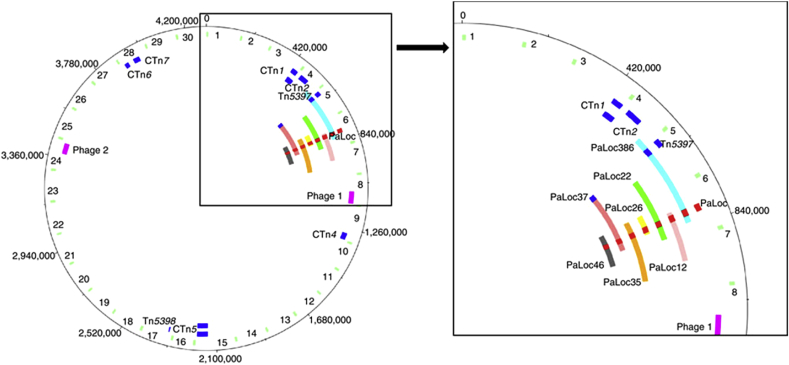
Demonstration of transfer of the PaLoc. The left panel represents the *C. difficile* 630 genome. The outer circle represents the *C. difficile* genome the green bands on the second circle represent 10 kb fragments around the 630 genome that contain snips and indels that differentiate the 630 donor genome from the CD37 recipient genome; the dark blue fragments on the third circle represents the location of CTns the red fragment on the third circle represent the location of the PaLoc, the purple fragments on the third circle represent the location of prophage. The fourth to the tenth circle represent CD37 transconjugants containing the PaLoc, transferred fragments are shown by coloured lines, transferred CTns and PaLocs are shown. The right hand panel shows the region containing the PaLoc in more detail. (for more details see reference [47]). Figure reproduced from; Brouwer MS, Roberts AP, Hussain H, Williams RJ, Allan E, Mullany P. (2013). Horizontal gene transfer converts non-toxigenic *Clostridium difficile* strains into toxin producers. Nature Communications. 4, 2601.

PaLoc transfer was initially discovered when selecting for a genetically marked copy of CTn*2* from *C. difficile* 630 to CD37. When investigating the transconjugants for co-transfer of non-selected genetic elements, it was found that one transconjugant also contained the PaLoc. Further transfer experiments with a donor bearing a PaLoc with an erythromycin resistance gene within *toxB* reproducibly yielded transconjugants and investigation of these showed that the PaLoc was transferred on variable sized genomic fragments ranging from 66 kbp to 272 kbp. This study also showed that the PaLoc was frequently co-transferred with CTns (Fig. 2) [61]. The transfer frequency of PaLoc and CTn was low, approximately 1 transconjugant per 10^8^ recipients however a non-selected CTn was almost always observed in transconjugants containing the PaLoc indicating that PaLoc and CTn transfer are linked. The exact mechanism behind this linkage is still under investigation however a hypothesis that explains these observations is that that one or more of the CTns provides an integrated origin of transfer for the transfer of large genome fragments containing the PaLoc. We propose that if a CTn does not excise, it may promote the transfer of large genomic fragments by CTaR.

Importantly, it was shown that the newly acquired toxin genes were expressed in the previously non-toxigenic strain, CD37, showing that non-toxigenic strains have the potential to be converted to toxin producers [61]. This is an important observation as non-toxigenic strains have been proposed as therapeutic agents for *C. difficile* disease [62], and there is an urgent need therefore to understand the factors promoting transfer of the PaLoc.

## 9 Conclusions

*C. difficile* contains a large number of MGEs and as we have discussed above, these have a wide-ranging effect on the biology of the bacterium. The major virulence factors of the organism, toxins A and B, are contained on a genetic island, the PaLoc, which is itself transferable on large genomic fragments, most likely mediated by the integrated CTns. In addition, bacteriophages can modulate expression of the toxin genes within the PaLoc. Thus, MGEs have a profound influence on the organism's pathogenicity. Further examples include the ability of Tn*5397* to insertionally target *C. difficile* genes containing Fic domains, which may have a role in virulence. MGEs also affect the biology of the organism in other ways: by spreading antibiotic resistance and other phenotypes enabling adaptation to stressful environments. The fact that some MGEs encode predicted sigma factors and other regulators indicates that these elements have the potential to modulate the regulatory pathways of *C. difficile* with, as yet unknown, but potentially profound consequences on the biology of the organism. Furthermore, the large number of MGEs in *C. difficile* and the genetic flexibility they afford undoubtedly contributes to the evolutionary success of this organism.

## Conflict of interest

The authors declare they have no conflict of interest.
